# A Statistical Framework for Person-centered Analysis of Digital Service Use in Public Health and Social Care

**DOI:** 10.1055/a-2844-0874

**Published:** 2026-04-18

**Authors:** Ilona Rönkkö, Antti Heiskanen, Katja Antikainen, Pia Liljamo, Ulla-Mari Kinnunen

**Affiliations:** 1 Department of Data and Analytics, Finnish Institute for Health and Welfare, Helsinki, Finland; 2 BCB Medical, Finland; 3 Wellbeing Services County of Kanta-Häme, Hämeenlinna, Finland; 4 Department of Health and Social Management, University of Eastern Finland, Kuopio, Finland; 5 Research Center for Nursing Science and Social and Health Management, Wellbeing Services County of North Savo, Kuopio University Hospital, Kuopio, Finland

**Keywords:** patient-centered care, data mining, registries, telemedicine, statistical models

## Abstract

**Background:**

Secondary use of health and social care registry data enables evaluation of service effectiveness. The focus is shifting from organization-based metrics to person-centered, system-level approaches that understand outcomes as the combined result of interacting actors. In this context, digital service use offers an important lens for understanding accessibility, participation, and equity within public services.

**Objectives:**

The aim of this study is to develop an interpretable analysis approach that uses a pre-existing person-centered, registry-based data model to identify individual variables associated with clients' digital service use.

**Methods:**

This registry-based study in a Finnish wellbeing services county applied a person-centered data model linking variables across health care, social services, and client experience. Digital service use was analyzed across 17 reason-for-encounter groups defined by the International Classification of Primary Care (ICPC) and one system-generated supplementary category arising from the dataset's internal structure. Subgroup analysis covered single and paired variables. Effect size was quantified using lift values from Market Basket Analysis, and generalizability was assessed aggregating uncorrected directional test outcomes across ICPC groups.

**Results:**

We developed a subgroup-based dual-loop analysis method to identify variable-specific associations across 18 ICPC categories. The method showed that digital service use was most common among younger individuals, women, and urban residents. Low service need and mental-health or family-center–related variables were associated with a higher likelihood of choosing a digital channel, whereas chronic illnesses, intensive care needs, and continuity of care were associated with a lower likelihood of digital channel selection.

**Conclusion:**

Registry data and a person-centered model reveal how individual variables influence digital service choices. The method enabled interpretable insights into single variables and their combinations. The findings support more equitable digital services and emphasize the need to tailor solutions to meet diverse client needs.

## Introduction

Health and social care systems face growing pressure to improve outcomes with limited resources, increasing the need to evaluate service effectiveness. The secondary use of health data enables more efficient health care and data-driven value solutions, but its success depends on the availability and quality of contextual data. 1 2 3 The emerging European Health Data Space (EHDS) is reshaping how data can be accessed and reused. 2 Improved data availability enables more systematic assessment of effectiveness and increases the need for frameworks and information requirements that guide such evaluation. 4 5 6 7 The World Health Organization (WHO) emphasizes data infrastructures that support personalized care and the purposeful secondary use of health information. 8 Effectiveness evaluation should be based on dynamic, system-level knowledge management that incorporates the citizen and client perspective and examines service processes through their overall impact on well-being, with individual visits or service episodes informing this broader evaluation. 9 10 11 12

Health and social care registries generate extensive and heterogeneous data, making structured, interoperable, and high-quality data essential for effective research. 3 Analytical choices must align with the structure and provenance of available data to ensure meaningful results, as modeling frameworks vary in suitability, assumptions, and interpretability, and many are not designed for heterogeneous registry data. 13 In this study, we selected an analytical approach suitable for registry-based data and transparent variable-level interpretation. We apply a structured association-analysis framework that supports the systematic assessment of individual-level variables and their combinations across the different service use contexts defined by reason-for-encounter categories.

Digitalization has become a strategic foundation for health and social care development. 14 The COVID-19 pandemic accelerated digital adoption, expanding the use of video consultations. 15 16 Before the pandemic, evidence on digital primary care focused mainly on telephone-based models with limited evaluation of effectiveness or patient needs. 17 Post-pandemic research shows that digital services expanded across population groups while digital inequality increased, particularly among older adults and socioeconomically disadvantaged populations. 18 Today, digital health services span video consultations, remote monitoring, the transmission of patient-generated data, online information seeking, and alert systems supporting continuity of care. 19 The WHO digital health intervention classification further systematizes this landscape by delineating capability-based categories that encompass targeted and untargeted communication, personal health tracking and self-monitoring, on-demand access to health information, and platform-based functionalities for reporting, financial transactions, and the management of consent. 20

Welfare technology must be assessed for its impacts on individuals, organizations, and society, with attention to ethical considerations and potential value conflicts. 21 Digitalization can streamline operational workflows, enhance patient care, and increase resource visibility and allocation efficiency; however, evidence shows that its impacts on population health, costs, and professional experiences vary substantially across clinical contexts. 22 23 24 25

In social services, digital solutions remain fewer and more fragmented, concentrated in areas such as home care. 15 Digital services can deepen inequality when disadvantaged users struggle with access, skills, or support; preventing inequities requires addressing digital health literacy, simplifying communication, and providing flexible alternatives. 18 26 27 28 Health and social care development emphasizes client-centered and outcome-oriented service structures, requiring dynamic knowledge management and person-centered data models. 9 10 12 In this study, “person-centered” refers specifically to data organized at the level of individuals as the units of analysis and does not imply alignment with experiential models of person-centered care.

Understanding the variables associated with digital service uptake supports more efficient and equitable service provision. Digital service users are primarily younger adults 29 30 31 32 33 and women. 30 31 32 Digital service users tend to have higher socioeconomic status, report high satisfaction, and be more prevalent in metropolitan areas. 29 30 31 34 35 Digital services are used for various health needs, such as digital visits for minor infections and in-person care for chronic conditions. 30 32 In this study, the use of digital services refers to the client's opportunity to choose between digital and traditional channels, and we examine which individual variables are associated with this choice across service contexts.

The aim of this study is to develop an interpretable analysis approach that uses a pre-existing person-centered, registry-based data model to identify individual variables associated with clients' digital service use. By focusing on variable-level associations within this established data structure, the study supports transparent examination of digital channel choices across diverse service-use contexts.

## Objectives

This study has the following objectives:

1. To identify an analysis method that, using registry-based data, can reveal which individual variables influence clients' use of digital services for health and social care.
2. To determine which individual variables identifiable through registry data are associated with clients' digital service use.

## Methods

### Exploratory Data-driven Study Design

The increasing availability of registry data on clients in public health and social care enables the formulation of research questions with greater flexibility. Instead of asking traditional hypothesis-driven questions such as “Does variable X affect phenomenon Y?,” it is possible to conduct exploratory analyses. This exploratory layer adds an important dimension to the study design: although the predictors of digital service use are established in the literature, exploratory analysis applied to the system-level data contained in a person-centered data model can reveal unexpected subgroups in which associations have not previously been reported or even suspected. Registry data provide population-level coverage, making exploratory approaches suitable for identifying subgroup patterns that are not visible in hypothesis-driven designs. This type of approach requires new methodological solutions and careful statistical planning, particularly to avoid pitfalls such as the multiple-comparisons problem. Because such questions do not yield a single value or test statistic as an answer but rather a broad and multidimensional set of results, key challenges include how to present, visualize, and interpret the findings in a meaningful way.

### Research Environment and Dataset

The study was conducted in a Finnish Wellbeing Services County (WSC), which has approximately 170,000 inhabitants. The statutory duties of the WSC include organizing health care, social, and rescue services. 36 Clients can access services through multiple digital and non-digital channels such as phone, chat, video, audio messages, letters, secure email, and digital platforms. Remote contacts are documented in the same way as face-to-face encounters. 37 38 The key digital services of the WSC under study include chat, video contacts, digital symptoms and health checkers, and periodic check-ups via a national digital service. In 2023 and 2024, the WSC's health care system recorded 177,839 unique clients and 7,902,175 contacts, of which 121,718 (1.5%) were digital. This client count includes individuals who used services from outside the WSC area or who no longer reside within it. Of these clients, 41,269 (23.2%) interacted digitally. Digital service use ranged from primary outpatient services (3.3%) to specialized hospital services (0.1%) and disability services (0%).

**Fig. 1:**
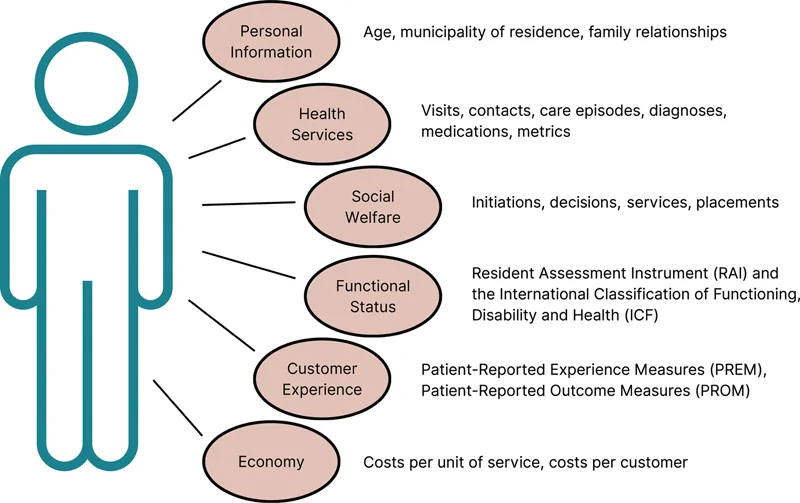
Examples of datasets within the dynamic person-centered data model. Source: Adapted from Antikainen et al. 40 Licensed under CC BY 4.0.

In this study, we utilized data from the years 2023 to 2024 originating from systems integrated into the WSC data lake. These included registry data from two social care information systems, primary and specialized health care systems (stored in two separate databases), digital service platforms, and data collected through the client-experience application. The dataset was structured using the existing WSC person-centered data model, which links pseudonymized information to individuals across health and social care sectors and served as the conceptual framework for defining the dataset. 39 40 This model enables system-level evaluation of demographic factors, health status, service use and needs, functional ability, client experience, and cost-related data. Fig. 1 illustrates the core concept of the model: an individual is associated with demographic, health-related, service-use, functional-ability, client-experience, and cost information.

**Table 1:** ICPC groups analyzed for proportions digital service use

A – General and Unspecified B – Blood, Blood Forming Organs and Immune Mechanism D – Digestive E – Eye H – Ear K – Cardiovascular L – Musculoskeletal N – Neurological P – Psychological R – Respiratory S – Skin T – Endocrine/Metabolic and Nutritional U – Urological W – Pregnancy, Childbearing, Family Planning X – Female Genital Y – Male Genital Z – Social Problems Common ICPC codes for special use (not an official international code)

Abbreviation: ICPC, International Classification of Primary Care.

A total of 171 variables were compiled and included information on personal background, such as age group, gender, municipality of residence, service use, service needs, client experience, and specific service-related details. To clarify how these constructs were operationalized, service use was defined as the number of digital and non-digital contacts recorded across service channels, professional groups, and organizational units. Service needs were captured using indicators reflecting clients' demand for services, including emergency clinic visits, contacts related to chronic conditions, diagnosis-based service-need coefficients, and the volume and diversity of social welfare–related encounters. Morbidity was assessed using ICPC reason-for-encounter groups, ICD-10 diagnostic categories, and select chronic disease groupings. Care-process and experience-related variables were identified through structured care-plan documentation and client-recommendation scores. Of the full variable set, 159 variables were numerical in nature (four indices and 154 counters), and 12 were categorical, with 73 combined levels among the variables. Thus, there were 232 different subgroups to which clients could belong. A detailed description of all variables is provided in Supplementary Table S1 (available in the online version only).

Some client data, such as age, area of residence, and certain indexes (e.g., continuity of care), were already available as single-value entries and required no further processing. Many descriptive variables were derived from contact records and converted into single-value descriptors using counters, which was appropriate because service need is not directly documented in the registry, whereas service use can be quantified reliably. Because several variables inherently reflect both service demand and service intensity, they were treated as dual-purpose indicators, a term used in this study to denote variables that simultaneously capture elements of need and intensity of use. This was appropriate for the exploratory design of the study, where the aim was to evaluate the association of each variable with digital service use rather than to isolate mutually exclusive constructs. Because variables were analyzed independently and not used in multivariable modeling, their dual role reduces the risk of introducing major statistical bias in this setting.

The data were structured into two components: a wide-format client-profile dataset (one row per client) and a service use dataset capturing system-level service events across both the health and social care sectors. In this service-use dataset, the key analytic variable was the proportion of visits conducted through a digital service, calculated separately for each reason-for-encounter group (International Classification of Primary Care, ICPC) as defined in the ICPC ( Table 1 ).

In the client-profile dataset, ICPC group-based variables were calculated as simple contact counts for each group. Because the analysis required variables suitable for segmenting client groups, numerical variables such as contact counts were assigned cutoff values. To ensure consistency across variables with varying prevalence, each cutoff was set at the median of nonzero values. If only a small proportion of clients, for example, 10%, had used a particular service, the cutoff value for that variable was determined based on the median of the nonzero values within that subgroup. This approach ensured that the above-median group included clients for whom the variable was meaningful, rather than those with only occasional or incidental contacts. The digital user status of each client was determined separately for each ICPC group, and the method of classification is described in the Method Selection section.

### Approach to Method Selection

The central methodological decision in this study is whether to adopt direct statistical modeling or to employ a more exploratory analytical approach. One of the key objectives is to obtain a comprehensive overview of digital service use, whereby each variable is assigned an interpretable and visualizable value that reflects the proportion of digital service use within the subgroup of WSC clients defined by that variable.

Two methodological paths are available: analyzing each variable independently or modeling all variables jointly. Several established modeling approaches, including PCA-based dimensionality reduction and logistic regression 41 as well as random forest classifiers, 42 have been applied widely in data-analytic and classification contexts. When modeling all variables jointly, methods must handle high dimensionality, multicollinearity, and heterogeneous variable prevalence. Among methods that consider all variables simultaneously, penalized regression methods are commonly used to address these challenges.

Given that the dataset includes both common and rare variables and that the total number of variables is considerable, penalized regression methods could, in principle, be used to model all variables simultaneously in a single framework. Penalized regression methods address high dimensionality and multicollinearity through shrinkage, but they do so by reducing coefficient magnitudes.

In lasso regression, shrinkage may set some coefficients exactly to zero, effectively performing variable selection, whereas ridge regression tends to diminish the effects of weak or correlated predictors. These shrinkage effects may obscure genuinely weak but meaningful predictors and complicate the interpretation of individual variable contributions. Because the aim of this study is interpretability rather than prediction, shrinkage-based multivariable models may not be the most appropriate choice for retaining the weak but directionally consistent signals that are central to understanding subgroup behavior.

Another modeling framework relevant for analyzing behavioral mechanisms is individual-based (agent-based) modeling, which simulates micro-level decision rules, interactions, and adaptive behavior. These models typically require explicit behavioral assumptions, detailed interaction structures, and high-resolution temporal data for calibration. Because our registry-based dataset consists of episodic service-use events rather than observable decision processes or interaction networks, the necessary inputs for agent-based modeling are not available. For this reason, we consider agent-based modeling an important but currently unsuitable approach for this dataset. 13

In the present context, the focus is on capturing the digital service use patterns of each subgroup. Although the variables are defined individually, many of them belong to broader hierarchical categories. For instance, variables representing chronic conditions are defined separately for each diagnosis; yet, if all chronic conditions exhibit small effects in the same direction, this collective pattern is important for understanding user profiles. Treating each variable solely as an independent predictor would cause multivariable shrinkage-based models to obscure these shared directional effects. Consequently, an analytical approach capable of examining or visualizing groups of related variables without excluding weak but potentially meaningful effects is required. For this reason, an approach in which all variables are analyzed separately, while the group structure remains visible in the visualizations, is adopted.

This choice introduces interpretive challenges, as the resulting estimates must be understood in the absence of adjustments for multicollinearity. Apparent effects in the results or visualizations may, therefore, be driven by unobserved, correlated variables. To address this limitation, an additional analytical step is included in which combinations of variables are examined. The objective of this complementary analysis is to assess whether the effect of a variable remains stable when paired with other variables, as detailed in the following section.

### Subgroup-based Dual-loop Analysis of Digital Service Selection

**Fig. 2:**
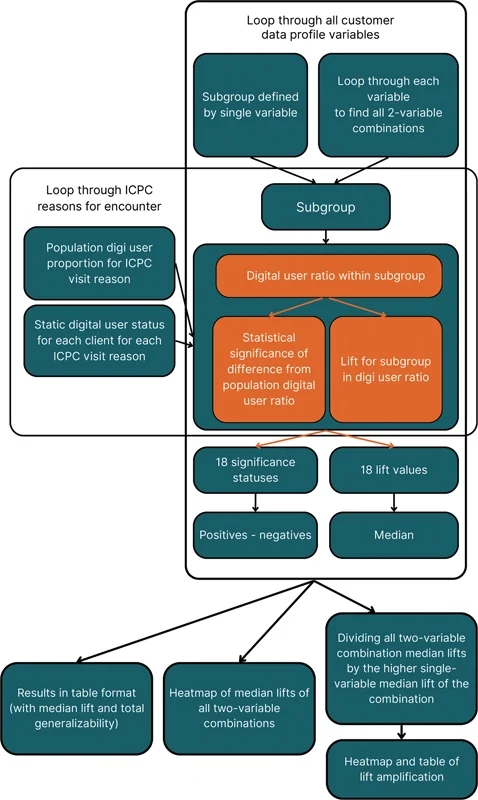
Overview of the analysis process.

The core idea of the analysis was to determine whether each variable was associated with a positive or negative effect on the selection of a digital service channel. To achieve this, a dual-loop analysis process was designed. An overview of this structure is presented in Fig. 2 and explained in more detail below.

The process begins with subgroup definition, enabling comparisons between clients within a subgroup and the overall population. In this study, subgroups were formed based on each individual variable as well as every two-variable combination. The process was repeated identically regardless of the basis for subgroups, and each variable was examined uniformly in both analysis groups. The analysis loop that iterated through all variables is illustrated in Fig. 2 , and in each round of the loop, a single subgroup was entered into the analysis (labeled “single variable analysis”).

Outside the loop, a static digital user status label was defined for each client for each ICPC group. Thus, each client entered the mathematical analysis with 18 different digital use statuses. A client was classified as a digital user if at least 10% of the client's visits within a given ICPC group were digital. This allowed for comparison of digital user proportions both within subgroups and relative to the entire client population (including both those inside and outside the subgroup). As shown in Fig. 2 , a population-level digital user proportion was calculated for all 18 ICPC groups, independent of the subgrouping based on person-centered variables. This served as a reference for comparative analysis.

Building on this structure, each variable and each two-variable combination were analyzed separately across the ICPC-based category set (17 ICPC groups plus one system-generated supplementary category). To conclude that a variable or variable combination was associated with the choice of a digital service channel, the association was required to appear in the same direction across multiple ICPC groups. This generalizability requirement reduced the risk that results would be driven by characteristics specific to a single ICPC group, for example, differences in digital-service availability, workflow practices, or care pathways. This approach ensured that the analysis reflected clients' actual channel-selection behavior rather than structural differences across service categories.

Once the subgroups had been defined and the statuses and reference values were calculated, the main analysis was conducted. For each subgroup, the proportion of digital users was calculated and compared with the ICPC group–specific reference value. To assess both effect size and significance or generalizability, two metrics were used: the lift value from Market Basket Analysis (MBA) (Eq. 1) and a generalizability quantity constructed from a set of non-Bonferroni-corrected significance statuses of the comparisons. This resulted in a temporary result set containing 18 lift values and 18 significance statuses, which are explained in more detail below. The median was used instead of the mean to reduce the influence of extreme subgroup proportions and ensure robust cross-group comparability.



(Eq. 1. Lift value from Market Basket Analysis)

Importantly, this calculation remained identical regardless of whether the subgroup was defined by a single variable or by a two-variable combination, as the lift depends only on subgroup size, population size, and digital-user proportions from both the subgroup and the overall population.

The generalizability metric is calculated from the set of 18 statistical significance test results. These results are not yet corrected for multiple comparisons, as they are only used as a dataset for generalizability. Each individual component of the generalizability metric is calculated using a basic statistical test to determine whether the digital user proportion within the subgroup is larger or smaller than that of the overall population at a statistically significant level. Instead of the actual *p* -value, this result is expressed as a counter, with each outcome representing positive significance, negative significance, or no significance.

After calculating these values for all 18 ICPC groups, the temporary result set was summarized into two final metrics to support visualization and interpretation. This approach was used to limit the volume of data from which conclusions were drawn, thereby enhancing the robustness of the analysis by reducing noise. Lift values were summarized using the median, whereas generalizability was calculated by subtracting the number of negative significances from the number of positive ones, yielding a scale from –18 to +18. As the null hypothesis would expect there to be no difference between the subgroup digital service user proportion and the overall digital service user proportion, making 18 comparisons and aggregating directional outcomes help reduce group-specific noise and increase directional consistency across ICPC groups; the metric is exploratory and not adjusted for multiple comparisons.

Despite these summarization steps, the result set remained large in terms of drawing conclusions, necessitating visualizations. Both single-variable and variable-combination results were tabulated, and key findings are presented in the Results section. To support comprehensive understanding, heatmaps were used. To reduce noise from rare combinations, only those present in at least 1% of the overall population or 1% of digital users were included. The generalizability metric was also applied as a filter.

The heatmap of variable combinations was based on the median lift value. Because lift is a ratio with a natural interpretive threshold at 1.0 (values below 1.0 indicate a negative effect and values above 1.0 a positive effect), a base-10 logarithm of the lift values was used. This ensured that the color scale emphasized meaningful differences and was centered around the interpretive turning point.

Variables in the heatmaps were grouped thematically (e.g., diagnoses, social services), with visual separators to aid interpretation of group-level effects. Diagonal intersections within groups may indicate cumulative effects (e.g., multiple chronic diagnoses), whereas cross-group intersections may highlight interactions (e.g., between health status and service use). The heatmaps also support interpretation at the level of individual cells, rows, or columns. For example, a row that is consistently redder than others suggests that the corresponding variable is a strong positive predictor. Cells that deviate from surrounding color patterns may indicate unique or unexpected interactions.

Because it is difficult to determine from a two-variable heatmap whether high values arise from the combination itself or from one strong individual variable, a separate heatmap was created. This visualization assessed whether the combination produced a higher median lift than either variable alone by dividing the combination's median lift by the higher of the two individual median lifts.

### Ethical Considerations

This study did not require ethical approval because it was based on the secondary use of pseudonymized registry data and did not involve any interventions or directly identifiable personal information. In Finland, the secondary use of social and health data is governed by the Act on the Secondary Use of Health and Social Data (552/2019), which regulates the use of personal data for purposes other than their original intent, such as research, development, and knowledge-based management. In registry-based research using pseudonymized data without direct identifiability or contact with individuals, ethical approval is generally not required, provided that the study does not involve interventions or the identification of individuals.

However, a data permit is always required when personal data are used. In this study, the data permit was obtained from the organization under study, as the data used originated solely from the registers of that specific WSC. In accordance with the Secondary Use Act, the data were processed in a secure environment, and the research results were anonymized before being transferred out of the processing environment.

## Results

The analysis applied a statistical framework that combines univariate association tests, select two-variable combinations, and an ICPC-based generalizability criterion. This framework provides a consistent basis for identifying variable-specific associations across different categories of digital service use and clarifies how the subsequent results were derived and evaluated.

### Impact of Age, Gender, and Municipality on Proportion of Digital Service Use

The proportion of digital service use was higher among younger age groups compared with the overall population ( Table 2 ). For example, the 18 to 24 age group had 98.9% higher use (median lift, hereafter MedLift, 1.989; net generalizability, hereafter NetGen 13), whereas the 75+ age group had 83.8% lower use (MedLift 0.162; NetGen –17). Gender also influenced usage. Women had 15.6% higher use (MedLift 1.156; NetGen 10), whereas men had 22.4% lower use (MedLift 0.776; NetGen –14). In Hämeenlinna, the largest municipality, use was 27.3% higher (MedLift 1.273; NetGen 13), whereas in Humppila, the smallest municipality, use was 55.3% lower (MedLift 0.447; NetGen –10).

**Table 2:** Effects of age, gender, and municipality on the proportion of digital service use

Variable category	Variable	Value	MedLift a	NegGen b	PosGen b	Unique *n* (subgroup) c	Median *n* across ICPC groups d
Person	Age group	0–17	0.805	−7	5	30,192	1,867
		18–24	1.989	−1	14	11,214	766
		25–64	1.499	0	16	55,248	3,479
		65–74	0.589	−13	0	22,391	1,597
		75–	0.162	−17	0	25,940	2,767
	Gender	Man	0.776	−14	0	66,370	4,655
		Woman	1.156	0	10	78,615	6,508
	Municipality	Outside the WSC	1.069	−5	3	11,261	554
		Loppi	0.921	−5	1	5,987	528
		Tammela	0.376	−14	0	4,587	289
		Jokioinen	0.396	−13	0	3,874	250
		Ypäjä	0.450	−9	0	1,666	100
		Hattula	0.344	−16	0	7,076	343
		Hämeenlinna	1.273	0	13	53,834	4,241
		Hausjärvi	1.022	0	2	5,957	467
		Humppila	0.447	−10	0	1,667	93
		Janakkala	0.862	−8	2	13,443	1,393
		Riihimäki	1.011	−5	5	22,476	1,602
		Forssa	0.581	−10	0	13,157	848

Abbreviations: ICPC, International Classification of Primary Care; WSC, Wellbeing Services County.

a MedLift (median lift across ICPC groups) summarizes the direction/strength of association with digital service use: values near 1 ≈ no effect; <1 negative; >1 positive.

b Generalizability across ICPC groups: PosGen counts groups with a positive (uncorrected) effect; NegGen counts groups with a negative (uncorrected) effect. NetGen = PosGen − NegGen, ranging from −18 to +18.

c Unique *n* (subgroup) = number of individuals in the respective subgroup.

d “Median *n* across ICPC groups” indicates the median number of unique individuals per ICPC group.

### Impact of Use of Social and Health Care Services on the Proportion of Digital Service Use

The top quartile of family center users ( Table 3 ) had 80.8% higher digital service use compared with the overall population (MedLift 1.808; NetGen 10). Similarly, users of mental-health and substance-abuse services had 30.3% higher use (MedLift 1.303; NetGen 7). In contrast, several services were associated with lower digital service use. The most pronounced decrease, of 95.4%, was observed among home-care users in the top quartile (MedLift 0.046; NetGen –18), followed by housing service users with an 85.0% decrease (MedLift 0.150; NetGen –18).

**Table 3:** Impact of service use modality on the proportion of digital interactions

Variable category	Variable	Value	MedLift a	NegGen b	PosGen b	Unique *n* (subgroup) c	Median *n* across ICPC groups d
Specialized care use	Usage	Above median	0.630	−7	0	301	29
	Contacts count	Non-zero occ. e	0.557	−7	0	426	42
Mode of health care service use	Total contacts	Top quarter	0.787	−12	2	43,596	5,764
	Primary contact channel	Consultation	0.428	−7	0	417	21
		Digital	9.825	0	18	1,752	132
		Home visit	0.180	−18	0	5,900	531
		In-person	0.938	−7	0	104,590	7,429
		Inpatient	0.000	−11	0	414	15
Distribution of professional non-digital contacts in health care	Proportion of all non-digital contacts	Doctors 50–75%	1.629	0	13	18,825	888
		Doctors >75%	3.019	0	12	1,756	48
		Other clinicians 50–75%	0.473	−15	1	4,451	221
		Other clinicians 25–50%	0.716	−8	0	4,896	248
		Other clinicians >75%	0.154	−16	0	3,026	165
		Nursing staff 25–50%	0.835	−10	0	22,235	2,366
Social service f	Services for the elderly	Non-zero occ.	0.000	−15	0	122	11
	Services for working age adults	Non-zero occ.	1.351	−1	7	2,251	195
	Family center services	Non-zero occ.	1.412	−2	9	3,015	329
	Substance abuse services	Non-zero occ.	0.629	−8	0	159	13
	Disability services	Non-zero occ.	0.473	−10	0	1,157	118
		Top quarter	0.093	−11	0	294	26
Health care service area	Housing services	Non-zero occ.	0.150	−18	0	3,836	371
		Top quarter	0.134	−15	0	904	93
	Specialized health care services	Top quarter	0.738	−13	0	24,712	3,048
	Home care	Non-zero occ.	0.163	−18	0	8,952	1,035
		Top quarter	0.046	−18	0	2,261	188
	Rehabilitation services	Non-zero occ.	0.712	−15	0	37,215	4,529
		Top quarter	0.628	−13	0	8,180	1,289
	Mental health and substance abuse services	Non-zero occ.	1.303	−2	9	14,320	1,324
	Primary health care outpatient services	Top quarter	0.862	−10	4	33,809	5,499
	Primary health care hospital services	Non-zero occ.	0.332	−16	0	9,316	999
		Top quarter	0.236	−16	0	2,253	274
	Family center services	Non-zero occ.	1.429	0	12	49,560	4,427
		Top quarter	1.808	−3	13	12,326	1,350
	Dental and oral health care services	Top quarter	0.907	−6	0	22,017	1,986
	Disability services	Non-zero occ.	0.000	−16	0	376	28
Digital service (Omaolo)	Dental/oral symptom/trauma, SC g	Non-zero occ.	3.980	0	10	232	21
	Health check for an 18-month-old	Non-zero occ.	2.659	-1	9	574	48
	Sexually transmitted disease, SC g	Non-zero occ.	3.571	−1	14	757	95
	Urinary tract infection, SC g	Non-zero occ.	3.430	−1	10	275	35
	General symptom checker, SC g	Non-zero occ.	5.614	0	17	1,850	228
		Top quarter	7.010	0	17	392	60
	Use of different checkers	Non-zero occ.	5.051	0	18	4,626	539
		Top quarter	6.371	0	17	541	101
	Total service usage count	Non-zero occ.	5.051	0	18	4,626	539
		Top quarter	5.888	0	17	988	151

a MedLift (median lift across ICPC groups) summarizes the direction/strength of association with digital service use: values near 1 ≈ no effect; <1 negative; >1 positive.

b Generalizability across ICPC groups: PosGen counts groups with a positive (uncorrected) effect; NegGen counts groups with a negative (uncorrected) effect. NetGen = PosGen − NegGen, ranging from −18 to +18.

c Unique *n* (subgroup) = number of individuals in the respective subgroup. Variables are reported when |NetGen| ≥ 6.

d ICPC = International Classification of Primary Care. “Median *n* across ICPC groups” indicates the median number of unique individuals per ICPC group.

e Non-zero occ. = non-zero occurrences.

f Subgroups with more than 100 unique individuals were included; in all other cases, a minimum of >200 was required.

g SC = Symptom Checker.

Use of specialized health care services above the median was associated with a 37.8% lower proportion of digital service use (MedLift 0.622; NetGen –7). The top quartile of health care contacts showed an even stronger negative association, with a 92.1% lower proportion (MedLift 0.078; NetGen –10). In contrast, digital contacts had a nearly 10-fold positive effect (MedLift 9.825; NetGen 18). Clients who had over 75% of their contacts directed to doctors had more than a 3-fold increase in digital service use (MedLift 3.019; NetGen 12), whereas contacts with other professionals were linked to lower digital use. Use of the digital service Omaolo, a CE-marked national platform in Finland providing symptom assessments and directing users to appropriate social and health services, showed a strong association with general digital service use, with MedLift values ranging from 2.659 to 7.010 and NetGen values from 8 to 18.

### Effects of Service Needs on the Proportion of Digital Service Use

Clients with low or occasional service needs ( Table 4 ) used digital services 28.4% more than the overall population (MedLift 1.284; NetGen 13). Digital engagement was comparatively lower among clients with complex needs. This included individuals receiving intensive multidisciplinary services (MedLift 0.741; NetGen –12), those assigned a disease-related weighting factor (MedLift 0.929; NetGen –6), and those falling within the highest quartile of emergency-service contacts (MedLift 0.910; NetGen –6). Social-service events were positively associated with digital use: initiation services (MedLift 1.216; NetGen 6), general social services (MedLift 1.218; NetGen 8), and income-support applications (MedLift 1.415; NetGen 9). In contrast, clients in the top quartile of placement services had 72.9% lower digital service use (MedLift 0.271; NetGen –10).

**Table 4:** Impact of certain factors describing the need for healthcare and social welfare services on the proportion of digital service use

Variable category	Variable	Value	MedLift a	NegGen b	PosGen b	Unique *n* (subgroup) c	Median *n* across ICPC groups d
Client segmentation e	Client group with a three-level classification	Low or occasional service need	1.284	−1	14	61,048	3,015
		Increased need for coordinated professional services	0.963	−3	0	64,199	5,429
		Intensive need for multidisciplinary or multi-agency services	0.741	−12	0	19,738	2,471
Disease-related weighting factor f	Diagnosis-based coefficient	Non-zero occ. g	0.929	−7	1	104,299	9,230
		Top quarter	0.708	−14	0	28,454	3,537
Need for emergency clinic services	Emergency clinic contacts	Non-zero occ.	0.910	−8	2	49,294	5,400
		Top quarter	0.909	−7	2	8,799	1,558
Service need related to social welfare	Initiation service	Non-zero occ.	1.216	−2	8	12,381	1,056
		Top quarter	1.309	0	5	2,650	236
	Social service	Non-zero occ.	1.218	−1	9	7,097	598
	Assessed need for different social welfare services	Non-zero occ.	1.061	−2	5	18,545	1,490
		Top quarter	1.038	−2	6	4,418	393
	Decision service	Non-zero occ.	1.035	−1	4	12,530	1,014
		Top quarter	0.965	−2	1	2,591	210
	Placement service	Top quarter	0.271	−10	0	344	33
		Non-zero occ.	0.614	−9	0	1,344	124
Applying for income support	Application activity	Non-zero occ.	1.415	−0	9	3,017	307
		Top quarter	1.371	−1	2	642	72

a MedLift (median lift across ICPC groups) summarizes the direction/strength of association with digital service use: values near 1 ≈ no effect; <1 negative; >1 positive.

b Generalizability across ICPC groups: PosGen counts groups with a positive (uncorrected) effect; NegGen counts groups with a negative (uncorrected) effect. NetGen = PosGen − NegGen, ranging from −18 to +18.

c Unique *n* (subgroup) = number of individuals in the respective subgroup. Variables are reported when |NetGen| ≥ 6.

d ICPC = International Classification of Primary Care. “Median *n* across ICPC groups” indicates the median number of unique individuals per ICPC group.

e The clustering model divides the WSC population into three groups based on overall interaction volume (e.g., visits) and whether this volume spans multiple care types or is concentrated in one or a few. The goal is to identify patients who could benefit from a personal doctor overseeing their care and those requiring multidisciplinary care coordination.

f Based on the national funding model for wellbeing services counties, which includes a coefficient for health care service needs.

g Non-zero occ. = non-zero occurrences.

Use of digital services is higher for the following reasons for encounter, diagnoses, or disease groups ( Table 5 ): pregnancy, childbirth, and the puerperium (MedLift 2.767; NetGen 15), female genital (MedLift 2.129; NetGen 16); neurotic, stress-related, and somatoform disorders (MedLift 1.540; NetGen 13); and mood disorders (MedLift 1.477; NetGen 10). Use of digital services is lower for general and unspecified reasons (MedLift 0.738; NetGen –7), blood and immune system issues (MedLift 0.284; NetGen –13), ischemic heart diseases (MedLift 0.332; NetGen –17), diabetes (MedLift 0.510; NetGen –17), and dementia (MedLift 0.142; NetGen –18).

**Table 5:** Impact of morbidity on the proportion of digital services use: Top five positively and negatively associated variables by category

Variable category	Variable	Value	MedLift a	NegGen b	PosGen b	Unique *n* (subgroup) c	Median *n* across ICPC groups d
ICPC reason-for-encounter groups	Female genital	Non-zero occ. e	2.129	0	16	4,045	600
	Pregnancy, childbearing, family planning	Non-zero occ.	2.462	0	15	9,104	816
		Top quarter	2.885	0	13	1,971	158
	Common ICPC codes	Non-zero occ.	1.781	0	13	2,791	466
	Respiratory	Top quarter	1.777	0	13	6,861	939
	Common ICPC codes	Non-zero occ.	0.787	0	13	2,787	464
	Blood, blood forming organs, and immune mechanism	Top quarter	0.284	−13	0	277	41
	Endocrine, metabolic, and nutritional	Non-zero occ.	0.758	−11	0	10,362	1,184
	General and unspecified	Top quarter	0.738	−10	3	25,739	2,902
	Endocrine, metabolic, and nutritional	Top quarter	0.629	−9	1	2,415	392
ICD-10 Diagnostic Groups f	Pregnancy, childbirth, and the puerperium	Non-zero occ.	2.767	0	15	2,891	216
		Top quarter	2.539	−1	8	689	58
	Mental, behavioral, and neurodevelopmental disorders	Non-zero occ.	1.147	−1	7	20,291	1,865
	Symptoms, signs, and abnormal clinical and laboratory findings, not classified elsewhere	Top quarter	0.814	−8	2	8,795	1,570
	Diseases of the circulatory system	Top quarter	0.326	−16	0	7,666	1,078
	Neoplasms	Non-zero occ.	0.624	−16	0	14,749	1,617
	Diseases of the circulatory system	Non-zero occ.	0.562	−15	0	31,336	3,559
	Diseases of the eye and adnexa	Top quarter	0.511	−15	0	3,161	413
	Neoplasms	Top quarter	0.331	−15	0	3,721	431
	Endocrine, nutritional, and metabolic diseases	Top quarter	0.590	−12	0	5,443	681
Chronic disease type g	Neurotic, stress-related and somatoform diseases, including eating disorders	Non-zero occ.	1.540	0	13	7,225	768
	Mood disorders	Non-zero occ.	1.477	0	10	6,048	637
	Epilepsy and migraine	Non-zero occ.	1.354	−1	9	4,864	577
	Neurotic, stress-related and somatoform diseases, including eating disorders	Top quarter	1.458	0	8	1,759	231
	Chronic diseases of the upper respiratory tract	Non-zero occ.	1.343	0	8	3,883	369
	Dementia and organic mental disorders g	Non-zero occ.	0.142	−18	0	4,640	492
	Cerebrovascular diseases	Non-zero occ.	0.283	−17	0	3,511	461
	Diabetes	Non-zero occ.	0.510	−17	0	11,223	1,245
	Ischemic heart diseases	Non-zero occ.	0.332	−17	0	4,988	574
	Cancer and in situ carcinomas	Non-zero occ.	0.349	−16	0	8,778	960

a MedLift (median lift across ICPC groups) summarizes the direction/strength of association with digital service use: values near 1 ≈ no effect; <1 negative; >1 positive.

b Generalizability across ICPC groups: PosGen counts groups with a positive (uncorrected) effect; NegGen counts groups with a negative (uncorrected) effect. NetGen = PosGen − NegGen, ranging from −18 to +18.

c Unique *n* (subgroup) = number of individuals in the respective subgroup. Variables are reported when |NetGen| ≥ 6.

d ICPC = International Classification of Primary Care. “Median *n* across ICPC groups” indicates the median number of unique individuals per ICPC group.

e Non-zero occ. = non-zero occurrences.

f ICD = diagnostic categories used to group patient conditions.

g Selected set of ICD-10 codes representing chronic conditions. The full list of selected ICD-10 codes is provided in Supplementary Table S2 (available in the online version only).

### Effects of Other Service-related Factors on the Proportion of Digital Service Use

Continuity of care was associated with lower digital service use ( Table 6 ). Clients in the top quartile of the doctors' continuity index had 72.9% lower digital use (MedLift 0.689; NetGen –9), whereas the nurse continuity index had an even stronger negative association (MedLift 0.590; NetGen –17). For other factors such as care plans and client satisfaction (derived from client-level mean recommendation ratings using NPS classification), no consistent association with digital service use was observed, as differences did not meet the threshold for generalizability.

**Table 6:** Impact of certain factors related to care and client experience on the proportion of digital services use

Variable category	Variable	Value	MedLift ^a^	NegGen b	PosGen b	Unique n (subgroup) c	Median *n* across ICPC groups d
Care coordination e	Care plan	No care plan	1.001	0	0	143,911	10,173
		Care plan created	0.938	−2	1	1,093	118
Continuity of care f	Doctors continuity index	Non-zero occ. h	0.812	−9	2	35,935	4,327
		Top quarter	0.689	−9	0	8,630	943
	Nurses continuity index	Non-zero occ.	1.003	−3	4	70,767	7,957
		Top quarter	0.590	−17	0	16,721	1,653
Client satisfaction	Client satisfaction (mean of 0–10 recommendation ratings per client for traditional services, NPS-based classification) g	Detractors	1.279	0	3	1,476	204
		Passives	1.058	−2	2	1,361	162
		Promoters	1.052	−7	4	20,017	1,883

^a^ MedLift (median lift across ICPC groups) summarizes the direction/strength of association with digital service use: values near 1 ≈ no effect; <1 negative; >1 positive.

b Generalizability across ICPC groups: PosGen counts groups with a positive (uncorrected) effect; NegGen counts groups with a negative (uncorrected) effect. NetGen = PosGen − NegGen, ranging from −18 to +18.

c Unique *n* (subgroup) = number of individuals in the respective subgroup.

d ICPC = International Classification of Primary Care. “Median *n* across ICPC groups” indicates the median number of unique individuals per ICPC group.

e A care plan was considered created if it was recorded in the patient information system with a structured content tag; additional details are provided in Supplementary Table S1 (available in the online version only).

f Continuity of care was assessed using a modified version of the Continuity of Care Index (COCI); see Supplementary Table S1 (available in the online version only).

g Classification is based on the client-level mean of all 0–10 recommendation ratings for traditional services during July–December 2024. For details, see Supplementary Table S1 (available in the online version only).

h Non-zero occ. = Non-zero occurrences.

### Joint Effect of Variable Combinations on Digital Service Use

Table 7 presents combinations of client-related variables and their joint effects on digital service engagement. The analysis introduces a variable that describes the ratio between the lift value (from Market Basket Analysis) of the variable combination and the higher of the lift values of the individual variables in the combination. This variable is referred to as “Ratio” in this article. This approach highlights cases where the combination is associated with higher digital use than either variable alone. The strongest relative effect was observed among clients with low or occasional service needs combined with diagnoses related to factors influencing health status and service contact (Ratio 6.519; MedLift 6.930; NetGen 6). Other notable combinations included clients aged 25 to 64 with general and unspecified ICPC-group diagnoses (Ratio 5.911; MedLift 3.693; NetGen 17), those using family-center services with musculoskeletal conditions (Ratio 5.618; MedLift 3.198; NetGen 9), and clients from Hämeenlinna with low nurse continuity (Ratio 2.828; MedLift 4.599; NetGen 17). Several combinations involving family-center services, musculoskeletal conditions, and continuity indices were consistently linked to elevated digital use, with younger age groups, particularly those aged 18 to 24 and 25 to 64, frequently appearing among the top-ranked combinations.

**Table 7:** Increase in digital service use beyond expected individual effects—Market Basket combinations

Individual variable 1 (category)	Individual variable 2 (category)	Ratio, lift of combination/highest individual lift a	Median lift b	Overall generalizability (NetGen) c	Unique *n* (subgroup) d	Median *n* across ICPC groups e
Factors influencing health status and contact with health services >Median—ICD	Low or occasional service need—Cluster	6.519	6.930	6	26,762	1,316
Age group 25–64—Person	General and unspecified >Median—ICPC group	5.911	3.693	17	31,075	2,146
General and unspecified >Median—ICPC group	Low or occasional service need—Cluster	5.688	1.622	9	41,706	1,961
Family center services >Median—Service area	Diseases of the musculoskeletal system and connective tissue >Median—ICD	5.618	3.198	9	7,500	881
Family center services >Median—Service area	Doctors continuity index >Median—Continuity	5.107	2.015	11	8,007	1,102
Family center services >Median—Service area	Rehabilitation services >Median—Service area	5.052	2.132	9	9,501	1,146
Diagnosis-based coefficient <Median—Disease-related weighting	Specialized health care services >Median—Service area	4.944	4.253	7	20,191	749
Rehabilitation services > Median—Service area	Age group 0–17—Person	4.204	4.093	6	5,590	404
Family center services >Median—Service area	Back diseases >Median—Chronic	3.959	3.517	9	2,727	315
Family center services >Median—Service area	Other musculoskeletal diseases >Median—Chronic	3.883	2.482	7	3,834	497
Doctor non-digital contacts High 50–75%—Professional	Nurses continuity index >Median—Continuity	3.602	4.212	16	6,989	541
Diseases of the circulatory system >Median—ICD	Age group 25–64—Person	3.418	3.647	12	9,114	623
Age group 25–64—Person	Ear >Median—ICPC group	3.082	3.206	6	3,557	316
Age group 25–64—Person	Hypertensive diseases >Median—Chronic	3.057	2.442	10	5,219	364
Municipality of Hämeenlinna—Person	Nurses continuity index <Median—Continuity	2.828	4.599	17	4,807	361
Municipality outside the WSC—Person	Nurses continuity index >Median—Continuity	2.699	2.600	9	2,916	237
Symptoms, signs, and abnormal clinical and laboratory findings, not elsewhere classified >Median—ICD	Age group 18–24—Person	2.625	3.623	14	2,556	266
Rehabilitation services >Median—Service area	Age group 25–64—Person	2.604	1.779	10	12,167	1,031
Doctor non-digital contacts High 50–75%—Professional	Musculoskeletal >Median—ICPC group	2.555	3.108	9	3,462	234
Eye >Median—ICPC group	Low or occasional service need—Cluster	2.548	3.004	6	3,201	161
Family center services >Median—Service area	Musculoskeletal >Median—ICPC group	2.525	1.734	11	8,875	1,101
Age group 18–24—Person	Nurses, continuity index >Median—Continuity	2.523	3.578	13	3,721	445
Family center services >Median—Service Area	Urological >Median—ICPC group	2.514	1.870	10	2,118	335

a This ratio shows how much more strongly the combination of two variables influences digital service use compared with the stronger of the two variables alone. Only combinations with a ratio ≥ 2.5 are reported. To ensure reliability, combinations must also have sufficient support (a large enough share of observations).

b Median lift calculated for the combination.

c Generalizability across ICPC groups: PosGen counts groups with a positive (uncorrected) effect; NegGen counts groups with a negative (uncorrected) effect. NetGen  =  PosGen  −  NegGen (range −18… + 18). Combinations were reported when |NetGen| ≥ 6.

d Median *n* across ICPC groups  =  the median number of unique individuals per ICPC group.

e ICPC = International Classification of Primary Care, reasons for encounter.

**Fig. 3:**
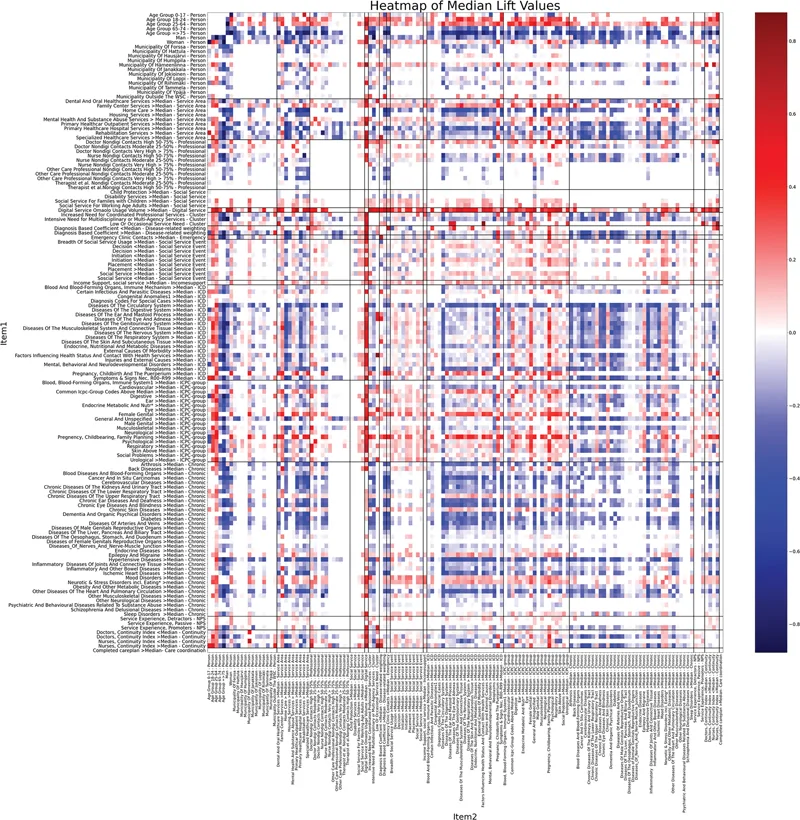
Heatmap of two-variable combinations from the Market Basket Analysis (MBA).

**Fig. 4:**
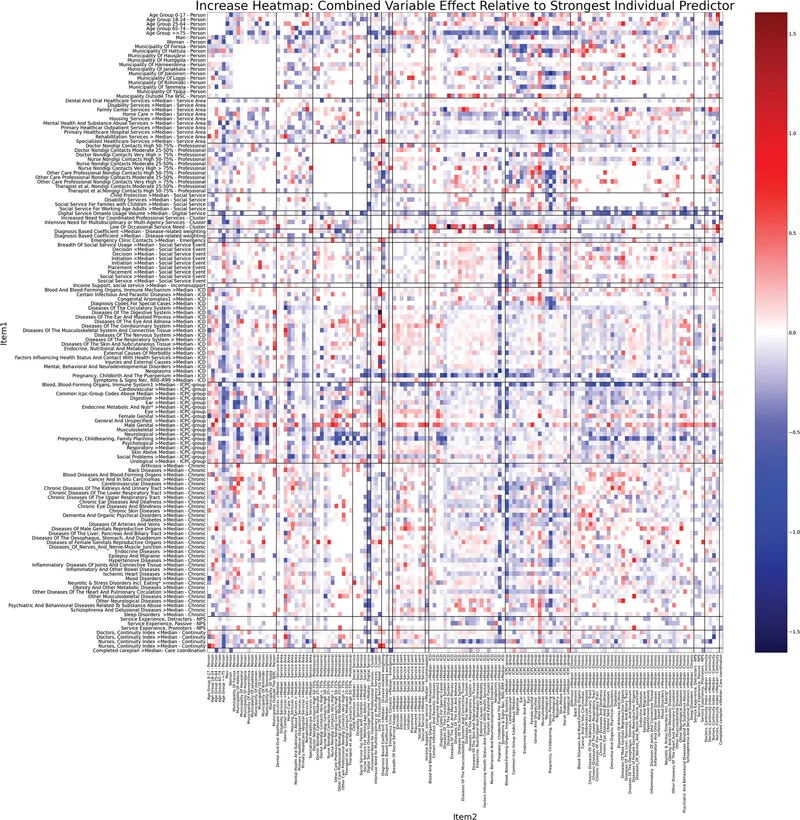
Heatmap illustrating the increase in lift due to the variable combination compared with the highest lift of the individual variables.

The study aimed to understand the factors influencing the selection of digital services and to develop a suitable methodology for this purpose. The heatmaps presented in the methodology section also serve as results, as they provide a visual overview of the effects of the variables. Fig. 3 presents a heatmap of two-variable combinations from the MBA analysis, and Fig. 4 presents a heatmap that illustrates the increase in lift due to the variable combination compared with the highest lift of the individual variables. The interpretations are not exhaustive but are intended to demonstrate how visual results can be interpreted according to the selected methodology.

One way to interpret the results is to focus on clear visual patterns in the usage heatmap ( Fig. 3 ). For example, increased use of social services consistently correlates with a higher probability of selecting digital services, regardless of other variables combined with it. This is visually represented as a light-red cross across the rows and columns labeled with social services.

Another set of clearly visible visual patterns includes other observable features, such as the low-service-need cluster, which is strongly associated with a higher likelihood of selecting digital services. Age groups also play a role: young adults are the most active users, and in the oldest age group, the selection of digital services is clearly lower. Additionally, home municipality has a visible impact; Hämeenlinna, the most urban area in the cohort, shows significantly higher digital service use than other municipalities. In contrast, chronic-illness diagnoses form a large blue area in the heatmap, indicating a generally negative association with digital service selection.

Further interpretation focuses on area-level interactions between grouped variables. In the heatmap, similar variables are organized into groups separated by black lines, allowing for the visualization of interactions between these areas, either within the same area or across different ones. For example, a predominantly red area representing a high number of visits within the same group suggests a large number of visits for various reasons, generally predicting a higher probability of selecting digital services. Another notable interaction is between visit frequency and social-service use, which also results in red areas, indicating a strong association with digital service selection.

The heatmap also highlights exceptional effects of individual variables. While most chronic illnesses are associated with reduced use of digital services, the heatmap shows that epilepsy, migraines, mental-health issues, and neurotic disorders, including eating disorders, deviate from this trend and are linked to higher use. These findings complement the results previously presented in tables and demonstrate how visual analysis can reveal meaningful details.

The Increase heatmap ( Fig. 4 ) provides a compelling perspective on how combinations of variables influence the selection of digital services. While the usage heatmap ( Fig. 3 ) shows that nearly all individual chronic illnesses are associated with reduced digital service use, the Increase heatmap reveals that combinations of two chronic illnesses often predict higher digital service selection than a single illness alone. A particularly noteworthy pattern emerges in the interaction between social-service use and the pregnancy, childbirth, and puerperium diagnosis group (ICD). The consistently blue coloring in the Increase heatmap suggests that although individuals with visits related to this diagnosis group are generally more likely to use digital services, those who also engage with social services tend to use digital services less.

## Discussion

This study set out both to identify suitable methods for uncovering the role of individual variables in digital service choices and to determine which specific variables, observable through registry data, influence those choices among health and social care clients.

### Methodological Considerations for Identifying Influential Variables

The selected methodology aimed to uncover the variables that are associated with the use of digital services. The chosen approach involved analyzing individual variables and their combinations separately, rather than using algorithms that first predict digital service use and then assess the influence of each variable, either by measuring the decrease in model accuracy when a variable is removed or by directly quantifying its contribution to the outcome. Because this method diverges from predictive modeling, its suitability for the original purpose is examined below. This step provided a methodological foundation suited for interpretable, registry-based variable analysis, consistent with the study's first objective.

The main advantage of this method, compared with predictive models, lies in the clarity of interpretation at both the micro and macro levels. Precise numerical values are available for each variable and combination, explaining their impact on digital service use. This provides a detailed view of small-scale phenomena, whereas large-scale effects can be visualized effectively using heatmaps. This effect is theoretically analogous to the ability of principal component analysis (PCA) to group similar variables into principal components. In addition to traditional multivariable and predictive modeling approaches, we also considered penalized regression techniques such as LASSO and ridge regression. Although these methods are effective in high-dimensional settings, their shrinkage-based estimation may suppress weak but meaningful associations, which is not aligned with this study's aim of retaining variable-specific signals across heterogeneous service categories. Likewise, individual-based (agent-based) modeling, although conceptually relevant for understanding behavioral mechanisms, requires explicit behavioral rules, interaction structures, and high-resolution temporal data that are not available in registry-based datasets. For these reasons, neither penalized regression nor agent-based modeling was deemed suitable for the present analytical framework.

From a scientific standpoint, the method does not account for the full client profile but focuses on individual variables. This enables equally accurate analysis of small subgroups compared with larger ones, even when the effect on digital service selection is driven by the larger groups, as individual-variable analysis does not suffer from variables being masked by stronger individual associations. This not only allows analysis of small subgroups behind stronger associations but also complicates interpretation, as the effect of a strongly associated variable on digital service selection is equally visible in all correlated variables. For this reason, all interpretations are presented as exploratory associations rather than causal conclusions, and correlated background variables are considered a potential source of observed patterns. The method does allow, through visualizations that display a large number of variable combinations simultaneously, the identification of correlated background variables, although this process is not straightforward.

One of the method's key applications is providing an overview of the variables associated with digital service use by summarizing a highly multidimensional and multilevel dataset into concise representations. Such summarizations always require a balance between what information is shown and what is omitted to ensure that key findings are communicated clearly and visualizations remain interpretable. Because this methodology preserves both small-scale and large-scale phenomena, albeit in a slightly different manner than predictive models, it should be interpreted within the context of exploratory analysis. The method is particularly well suited for identifying interesting subgroups that warrant more detailed analysis or investigation to uncover deeper relationships.

This study introduces a novel analytical approach that combines univariate association analysis with a systematic two-variable combination procedure and an ICPC-based generalizability requirement. This framework enables the identification of interpretable, variable-specific patterns from heterogeneous registry data without relying on multivariable modeling assumptions. The approach also highlights combinations of individual characteristics that consistently relate to digital service use across multiple service categories, offering a transparent way to examine complex population-level behaviors using routine registry data.

### Clinical and Public Health Implications for Health and Social Services

The findings aligned with previous evidence, indicating that, at an exploratory level, women, 30 31 32 younger individuals, 29 30 31 32 33 and urban residents 34 are more likely to use digital services. Throughout the article, the terms “complex needs” and “high needs” refer to clients with intensive or multidisciplinary service use. Digital users had lower overall service needs and lower utilization, whereas higher needs and strong care continuity were associated with reduced digital engagement. The use of digital services was more strongly associated with simple matters and individuals with minimal health concerns, especially in the low-need segment. 30 32 These findings are meaningful in light of previous research showing that digital health services support patient engagement and self-care. 19

In the context of social services, the findings were mixed. While the use of family services and certain processes, such as submitting an income-support application, was associated with a higher proportion of digital service use, other results indicated that digital engagement among users of social services was, to some extent, lower. Based on the analysis of combined variables, the use of social services consistently correlated with a higher likelihood of selecting digital services, which supports previous findings related to specific processes. On the other hand, increased use of social services in combination with pregnancy and childbirth diagnoses appeared to reduce digital service use. This association may be explained by the typically greater need for in-person support in this combination of circumstances. However, the ICPC-based generalizability requirement reduces the risk that the observed result would be driven by the characteristics of a single service category.

Previous studies have shown that the digitalization of social services has lagged behind health care. 15 Conversely, digital service users typically come from higher socioeconomic backgrounds. 29 30 31 Despite this variation, it is crucial to assess whether digital solutions meet the needs of high-need populations and avoid reinforcing service inequities. 18 19 26 27 28

Finally, the findings can support the development of more impactful and equitable digital services. Previous studies have emphasized that development efforts should take into account a broad range of needs and impacts, including individual, societal, ethical, and economic benefits. 21 To increase usage, it is important to identify which digital solutions best serve clients with more intensive service needs. 17 In some cases, digital services can complement traditional ones, requiring adjustments in service processes and for professionals to operate across both channels. The findings also enable more detailed analysis, for example, among people with diabetes, where self-care is emphasized yet digital service use is lower compared with the general population, indicating untapped potential. These associations should nevertheless be interpreted cautiously, as alternative explanatory variables, such as clinical guidance, resource availability, or service configuration, may also contribute to the observed patterns.

In contrast, mental-health service users show higher digital engagement, suggesting that expanding digital options could improve efficiency and reduce access barriers, especially when individual and organizational resources are limited. The combined-variable analysis revealed exceptions among chronic illnesses: although most conditions were associated with lower digital service use, diagnoses such as epilepsy, migraines, mental-health issues, and neurotic disorders, including eating disorders, showed higher levels of digital engagement. This suggests that expanding digital services within certain diagnostic groups could be particularly impactful. Furthermore, the Increase heatmap view of the analysis showed that a combination of two chronic illnesses increased the likelihood of using digital services compared with a single condition alone, suggesting at an exploratory level that multimorbidity is not necessarily a barrier to digital participation. These findings are exploratory and should not be interpreted as causal evidence but rather as signals indicating areas where further targeted research is warranted.

### Perspectives on Registry-based Study and Impact Evaluation

This section explores how a person-centered data model influences research, enables the secondary use of health data, and supports the development of impact evaluation. Current trends emphasize the utilization of accumulated social and health care data for the benefit of clients. 8 This study suggests that registry data can provide detailed insights into individual and contextual variables influencing service choices. The person-centered model supports a strategic shift toward client-oriented service development. 9 10 12 The data model enabled the integration of demographic, service use, and experiential data at the individual level. This supported a holistic examination of digital service use at both the individual and system levels. In addition, the applicability of the model in secondary-use contexts, such as service-system development and impact assessment, proved promising. Although not all dimensions of the model could yet be utilized (for example, data on functional capacity and costs were unavailable), the study laid a foundation for further development and broader data-driven decision-making.

The findings of the study contribute to the broader discussion on the evolving role of health data in service development and impact evaluation. The World Health Organization (WHO) emphasizes the need to build infrastructures that support personalized care and enable the secondary use of health data. 8 The person-centered data model used in this study reflects this direction, allowing the integration of experiential and demographic data to support dynamic knowledge management. 9 10 11 The model's potential for secondary use, such as in service-system development and impact evaluation, also aligns with the goals of the Value-based Health Care framework and the Quadruple Aim model, which emphasize client-centeredness, system efficiency, and professional well-being. 5 6 7

When considering broader implications, future development should focus on strengthening the model's capacity through the inclusion of new data sources. For example, digital services increasingly enable clients to provide self-reported information about their health and well-being. This enriches the understanding of the client's own perspective and lived experience, which is essential for meaningful impact evaluation. Enhancing the model in this direction would improve the ability to assess service responsiveness, identify gaps, and tailor digital solutions to different population groups. Furthermore, the registry-based framework offers a scalable foundation for impact evaluation, enabling the assessment of interventions not only at the individual level but also at the system level.

### Limitations

The analytical scope of this registry-based study was defined by the structure and availability of system-recorded data. Although a person-centered data model was used, which enabled system-level analysis of client-linked data across different systems, social care data were more limited in scope than health care data because of differences in information systems and documentation practices. However, this did not prevent the expansion of the analysis to the system level based on the available data.

Data available for secondary use in health and social care currently contain relatively little client-generated information. However, this type of data is increasing because of the opportunities enabled by digital services. In the future, such information may enrich the understanding of a client's situation, including aspects such as socioeconomic circumstances, competencies, or attitudes of the client.

The interpretation of certain variables, such as care-plan documentation, may be influenced by organization-specific practices. This means that the same variable may not represent the same concept across different organizations.

The person-centered data model is still under development, and not all of its potential dimensions could be utilized in this study. For example, data on functional capacity and costs were not yet available for analysis.

The study produced a large number of detailed findings. Therefore, the results cannot be directly translated into precise conclusions for service development. Instead, they must be interpreted and analyzed in relation to the particular organization's service portfolio and objectives.

In this article, the results were analyzed only at the population-wide level. However, the method allows similar analysis of subpopulations. For example, age group–specific heatmaps could support more direct conclusions for digital-service development. Some of the current findings may be influenced by certain services being used only by specific age groups, whereas the reference population includes the entire study dataset.

## Conclusion

This study demonstrates the value of registry-based data structured through a person-centered model in understanding digital service use at the system level. The findings call for more inclusive data ecosystems that incorporate client-generated information to better capture individual needs and contexts. Transparent and interpretable methods are essential for guiding ethical and equitable digital transformation. Continued development of data models and the expansion of data dimensions will be key to advancing data-informed planning and enhancing impact evaluation in health and social care.
