# Supplementary material for: A Statistical Framework for Person-centered Analysis of Digital Service Use in Public Health and Social Care
Source: Methods Inf Med. 2026 Apr 18;64(03-06):110–28. doi: 10.1055/a-2844-0874 (PMC13441484; doi:10.1055/a-2844-0874)
Supplement: Supplementary file 1 [file 10-1055-a-2844-0874-s25010135.pdf]

Supplementary Material

Related to the manuscript: A Statistical Framework for Person-Centered Analysis of Digital Service Use in Public Health and Social Care, Authors: Ilona Rönkkö, Antti Heiskanen, Katja Antikainen, Pia Liljamo, Ulla-Mari Kinnunen

Introduction

This supplementary material provides structured definitions of dataset variables and diagnostic classifications that underpin the analytical framework presented in the manuscript A Statistical Framework for Person-Centered Analysis of Digital Service Use in Public Health and Social Care. Its purpose is to support methodological transparency and facilitate reproducibility. Table S1 outlines the variable architecture used in the person-centered data model, while Table S2 presents ICD-10 code groupings for chronic conditions, adapted from Wikström et al. (2023).

Supplementary Table S1 Dataset variable structure and descriptions

Category / Component of person-centered data model	Type	Variable (if categorical), Index/counter (if numerical)	Levels / Variables	Additional context (data collected during 2023–2024 unless otherwise stated)
Person / Personal information	Categorical	Municipality	12	The client's municipality of residence.
	Categorical	Gender:<br>Male<br>Female	2	The client's gender
	Categorical	Age group:<br>0–17<br>18–24<br>25–64<br>65–74<br>75+	5	The client's age group (static throughout the study, i.e. no effect of aging during study period was included)
Care coordination / Health services	Numerical	Counter	1	The amount of recorded care plans in the patient information system with a structured content tag.
Specialized care use /	Numerical	Counter	5	Total number of special health care contacts

Health services				grouped by service channel (phone, digital, visit, other) and total amount
	Categorical	Usage	2	Total use of specialized hospital services categorized as above or below the median based on contact volume.
Continuity of care / Health services	Numerical	Index (for doctors and nurses).	2	The index is calculated by squaring the number of visits to each provider, summing these values, subtracting the total number of visits, and dividing the result by the square of the total number of visits minus the total number of visits. The index is based on the Continuity of Care Index (COCI) [1-2]. The COCI index reflects how consistently a client sees the same provider. Higher

				values mean care is more concentrated with fewer professionals. Departing from standard definitions of the COCI index, in this study the index was calculated to include not only non-urgent but also urgent contacts in primary care, due to data-related constraints.
Mode of healthcare service use / Health services	Numerical	Counter	9	Total number of contacts grouped by recorded contact types.
	Categorical	Contact intensity	2	Status variables on whether the overall number of contacts is higher or lower than the overall median
	Categorical	Most frequent contact type	7	The contact type with the highest number of recorded interactions per individual, based on contact categories.

	Categorical	Interaction category combined	11	Typical contact type combined with digital contact status.
	Categorical	Interaction category	6	The client's most frequent contact type, categorized based on recorded contact types.
	Categorical	Digital contact intensity	2	Status variables on whether the overall number of digital contacts is higher or lower than the overall digital contact amount median
The International Classification of Primary Care (ICPC) Reason-for-encounter groups/ Health services	Numerical	Counter	18	Classification is based on 17 reason-for-encounter groups defined by the International Classification of Primary Care (ICPC), and one additional group labeled 'Common ICPC codes for special use', which is not part of the official international classification, but are

				commonly used in Finnish health information systems and service analytics.
Distribution of professional non-digital contacts in healthcare/ Health services	Numerical	Counter	5	Number of non-digital contacts provided by each professional group.
	Categorical	Dominant group and proportion	18	Dominant professional group providing services and the concentration level of their contacts (e.g. “Nurses: High concentration (50%–75%)”).
Need for emergency clinic services / Health services	Numerical	Counter	1	Number of emergency clinic contacts recorded for each client.
Chronic condition / Health services	Numerical	Counter	35	Number of visits per client for selected chronic disease groups, based on ICD-10 code prefixes.

				Derived from the study "Multimorbidity transitions and the associated healthcare cost among the Finnish adult population"[3]. Each column shows the number of visits related to the corresponding disease group. The values are calculated by checking the presence of relevant codes per person.
Disease-related weighting factor / Health services	Numerical	Index	1	Index based on ICD-10 diagnoses estimating an individual's healthcare service need. The coefficient is aligned with the national funding model for Wellbeing Services Counties (WSC), where diagnosis-based weights influence the allocation of state funding[4].

International Classification of Diseases (ICD-10) Diagnostic Groups / Health services	Numerical	Counter	23	Number of visits per individual grouped by ICD-10 disease chapter (only visits that are documented as relating to the diagnoses are included)
Service area of healthcare / Health services	Numerical	Counter	11	Number of contacts per individual grouped by organizational service areas.
Service need related to social welfare / Social welfare	Numerical	Counter	5	Number of distinct events per individual for four social service types: initiation, placement, decision, and service contact. The derived variable different services (0–4) indicates how many of the following event types occurred for the individual: initiation, placement, decision,

				service contact (maximum 4).
Social service / Social welfare	Numerical	Counter	9	Number of contacts for each service category.
Applying for income support / Social welfare	Numerical	Counter	1	Number of applications for supplementary or preventive income support submitted by a client.
Digital service Omaolo / Independent data of the data model	Numerical	Counter	32	Number of times each Omaolo [5] service was used, along with two summary variables representing total Omaolo usage.
Client segmentation / Health services and social welfare	Categorical	Clusters:<br><br>Increased Need for Coordinated Professional Services	3	The WSC population is clustered based on the number of service interactions (e.g., visits) and whether these interactions are concentrated in one or a few types of care or

		Low or Occasional Service Need<br><br>High Complexity Requiring Multidisciplinary Coordination.		spread across multiple types. The goal is to identify clients who may benefit from either a dedicated physician overseeing their care or coordinated support from a multidisciplinary team.
Client experience / Client experience	Numerical	Average recommendation rating (0–10) [5]	1	Mean of all 0–10 recommendation ratings per client during July–December 2024 for traditional (non-digital) services only; higher values indicate greater willingness to recommend services.
	Categorical	Client Category:<br>Promoters<br>Passives<br>Detractors	3	Classification based on the client-level mean during July–December 2024 for traditional (non-digital) services only: Detractor: 0–<7, Passive: 7–<9, Promoter: 9–10. Classification follows the

				Net Promoter Score (NPS) [6] framework based on average ratings for traditional services.
Total number of distinct variables			232	

1 Finnish Institute for Health and Welfare (THL). Continuity of care (COCI) in primary health care nurse visits. Sotkanet.fi – Statistics and Indicator Bank. Indicator 5503. Updated April 14, 2025. Accessed August 18, 2025. https://sotkanet.fi/sotkanet/en/metadata/indicators/5503

2 $$\text{COCI} = \frac{\sum_{j=1}^{M} n_j^2 - N}{N(N-1)}$$

Where:

M = total number of providers

n_j = number of visits to provider j

N = total number of visits

COCI is a widely recognized international index. This formulation follows the general definition used by the Finnish Institute for Health and Welfare; see reference [1] above.

3 Wikström K, Linna M, Reissell E, et al. Multimorbidity transitions and the associated healthcare cost among the Finnish adult population during a two-year follow-up. J Multimorb Comorb. 2023;13:1-9. doi:10.1177/26335565231202325

4 Ministry of Finance, Finland. Funding of wellbeing services counties. Updated 2025. Accessed May 29, 2025. https://vm.fi/en/funding-of-wellbeing-services-counties

[5] Omaolo is a national digital service for social welfare, health care, and oral health in Finland. It supports self-care and directs users to appropriate services through symptom assessments, health checks, and wellbeing coaching. CE-marked under EU MDR 2017/745, it ensures high quality and safety. The service is developed and maintained by DigiFinland with municipalities, hospital districts, and technology partners. More info: https://digifinland.fi/en/our-operations/omaolo-service/"

[6] Bain & Company. Measuring Your Net Promoter Score. Net Promoter System. Available from: https://www.netpromotersystem.com/about/measuring-your-net-promoter-score/. Accessed August 18, 2025

Supplementary Table S2 Chronic conditions and selected ICD-10 codes (adapted from Wikström et al., 2023).

**Disease group**	**ICD-10 codes**
Arthrosis	M15- M19
Back diseases	M40-M43, M45-M51, M53-M54
Blood diseases and blood-forming organs	D50-D53, D55-D61, D63-D64, D66-D69, D71-D72, D73-D77, D80-D84, D86, D89
Cancer and in-situ carcinomas	C00-C26, C30-C34, C37-C41, C43, C44-C58, C60-C85, C88, C90-C97, D00-D07, D09
Cerebrovascular diseases	I60-I69
Chronic diseases of the kidneys and urinary tract	N01, N03-N05, N07-N08, N11, N18-N22, N31-N33, N35

Chronic diseases of the lower respiratory tract	J41-J47, J60-J68, J70, J84, J92, J95-J96, J98- J99
Chronic diseases of the upper respiratory tract	J30-J34, J35, J37-J38
Chronic ear diseases and deafness	H80-H83, H90-H91, H93, H95
Chronic eye diseases and blindness	H17-H21, H25-H28, H31, H33, H35-H36, H40, H42, H47-H49, H51, H54
Chronic skin diseases	L10, L12-L13, L20-L21, L23-L24, L26, L28, L40-L41, L43-L45
Dementia and organic mental disorders (*originally referred to as 'psychical'*)	F00-F07, F09, G30-G32
Diabetes	E10-E14
Diseases of female genitals/reproductive organs	N80-N81, N88
Diseases of male genitals/reproductive organs	N40-N42, N48, N50
Diseases of nerves and nerve-muscle junction	G50-G60, G62-G64, G70-G73
Diseases of the liver, pancreas, and biliary tract	K70-K77, K80-K81, K86
Diseases of the oesophagus, stomach, and duodenum	K21-K23, K25-K31
Diseases of arteries and veins	I70-I73, I78-I79, I83-I87, I89
Endocrine diseases	E00-E03, E05-E07, E20-E29, E31, E34-E35
Epilepsy and migraine	G40-G41, G43-G44
Hypertensive diseases	I10-I13, I15, I95, I97-I99

Inflammatory and other bowel diseases	K50-K52, K55, K57-K59, K62-K63, K90
Inflammatory diseases of joints and connective tissue	M05-M14, M30-M36
Ischemic heart diseases	I20-I25
Mood disorders	F30-F34, F38-F39
Neurotic, stress-related and somatoform diseases, including eating disorders	F40-F42, F44-F45, F48, F50, F52-F55, F59-F63, F68-F69
Obesity and other metabolic diseases	E65-E66, E68, E70-E72, E74-E80, E83-E85, E88- E90
Other diseases of the heart and pulmonary circulation	I27-I28, I31, I34-I39, I42-I45, I48-I51
Other musculoskeletal diseases	M20-M24, M70, M72-M73, M75, M79-M84, M91, M93-M94, M96, M99
Other neurological diseases	G10-G13, G20-G26, G35, G37
Psychiatric and behavioral diseases related to substance abuse	F10-F16, F18-F19
Schizophrenia and delusional diseases	F20-F22, F24-F25, F28-F29
Sleep disorders	F51, G47

Source: Wikström K, Linna M, Reissell E, et al. Multimorbidity transitions and the associated healthcare cost among the Finnish adult population during a two-year follow-up. J Multimorb Comorb. 2023;13:1-9. doi:10.1177/26335565231202325
